# Transcriptional regulation of proanthocyanidin biosynthesis pathway genes and transcription factors in *Indigofera stachyodes* Lindl. roots

**DOI:** 10.1186/s12870-022-03794-4

**Published:** 2022-09-13

**Authors:** Chongmin Wang, Jun Li, Tao Zhou, Yongping Zhang, Haijun Jin, Xiaoqing Liu

**Affiliations:** Guizhou University of Traditional Chinese Medicine, Guiyang, 550025 China

**Keywords:** *Indigofera stachyodes*, Proanthocyanidins, Biosynthesis, Transcriptome, Transcription factors

## Abstract

**Background:**

Proanthocyanidins (PAs) have always been considered as important medicinal value component. In order to gain insights into the PA biosynthesis regulatory network in *I. stachyodes* roots, we analyzed the transcriptome of the *I. stachyodes* in Leaf, Stem, RootI (one-year-old root), and RootII (two-year-old root).

**Results:**

In this study, a total of 110,779 non-redundant unigenes were obtained, of which 63,863 could be functionally annotated. Simultaneously, 75 structural genes that regulate PA biosynthesis were identified, of these 6 structural genes (*IsF3′H1, IsANR2, IsLAR2, IsUGT72L1-3, IsMATE2, IsMATE3*) may play an important role in the synthesis of PAs in *I. stachyodes* roots. Furthermore, co-expression network analysis revealed that 34 IsMYBs, 18 IsbHLHs, 15 IsWRKYs, 9 IsMADSs, and 3 IsWIPs hub TFs are potential regulators for PA accumulation. Among them, IsMYB24 and IsMYB79 may be closely involved in the PA biosynthesis in *I. stachyodes* roots.

**Conclusions:**

The biosynthesis of PAs in *I. stachyodes* roots is mainly produced by the subsequent pathway of cyanidin. Our work provides new insights into the molecular pathways underlying PA accumulation and enhances our global understanding of transcriptome dynamics throughout different tissues.

**Supplementary Information:**

The online version contains supplementary material available at 10.1186/s12870-022-03794-4.

## Introduction

Proanthocyanidins (PAs) are the polymers or oligomers of flavan-3-ol units, usually epicatechin (EC) and catechin (C), and are widely distributed in grape seed and tea plant. In the current research progress, PA extract has a variety of medical values, and can be used for anti-aging, prevention of cardiovascular and tumor, etc [1]. According to the types of flavan bonds, PAs are mainly divided into A-type and B-type. Of which B-type PA is the most frequent found in plant kingdom, their constitutive units are singly linked by C4–C8 or C4–C6 bonds, such as procyanidins B1, B2, B3, and B4 [1–3].

The biosynthesis of PAs is a part of the flavonoid pathway that has been well-characterized over the past two decades with the identification of numerous structural, regulatory, and transport-related genes [1, 4, 5]. Genes involved in each biosynthetic step from phenylalanine to flavan-3-ols (( +)-catechin and ( −)-epicatechin) have been well characterized, including phenylalanine ammonia lyase (PAL), cinnamate-4-hydroxylase (C4H), 4-coumarate ligase (4CL), chalcone synthase (CHS), chalcone isomerase (CHI), flavanone 3-hydroxylase (F3H), dihydroflavonol reductase (DFR), flavonoid 3′ hydroxylase (F3′H), anthocyanidin synthase/leucoanthocyanidin dioxygenase (ANS/LDOX), anthocyanidin reductase (ANR) and leucoanthocyanidin reductase (LAR). The synthesis of PAs and anthocyanins share common steps leading to flavan3,4-diols (such as leucoanthocyanidin), which can be converted to catechin (2,3-trans-flavan-3-ol) by LAR [6] or to anthocyanidin by ANS [7, 8]. Anthocyanidin then either serves as the substrate for the synthesis of epicatechin (2,3-cis-flavan-3-ol) by ANR [9]. Flavan-3-ol precursor will be glycosylated and transferred to vacuoles for polymerization. It is clear that epicatechin is glycosylated to form epicatechin 3′-O-glucoside with the participation of UDP-glycosyltransferase (UGT72L1), and then epicatechin-3′-O-glucoside is translocated into the vacuole via specific transporters of the multidrug detoxification and extrusion (MATE) factor family [10, 11]. However, the details of the polymerization process controlled by TT10(LAC15) in vacuole are still unclear [12]. Moreover, these pathway structural genes are regulated by a variety of transcription factors (TFs). To date, TFs of R2R3-MYB [13], bHLH [14, 15], WD40 [16], WIP [17], MADs [18], and WRKY [19, 20] families have been found to regulate PA biosynthesis. Among them, MYB TFs play a key role in the regulation of PA biosynthesis.

*Indigofera stachyodes* Lindl (*Papilionoideae* family) is distributed mainly in Guizhou, Yunnan, and Guangxi provinces. Its roots were known as Xuerenshen in Chinese and commonly used as the Miao traditional medicine for the treatment of cold fever, cough, etc. The distinguishing feature of *I. stachyodes* is its "blood" (i.e., it is reddish-brown after root bark is scraped off). This phenomenon is affected by many internal and external factors, but flavonoids content and type are among the most important factors that determine root color [21]. Flavonoids will gradually accumulate as the plant grows, and roots over three years old are regularly regarded as the harvesting standard of medicinal materials [22, 23]. Previous phytochemical studies indicated the presence of over 30 compounds in *I. stachyodes*, including epicatechin, stigmasterol, stigmast-4-en-3-one, l-maackiain, etc. [21, 24–26]. In the current research, we studied the flavonoid composition in *I. stachyodes*, and further found that procyanidin B2, catechin (C), epicatechin (EC), and epicatechin gallate (ECG) were the main flavonoids in *I. stachyoides* roots [27]. The flavonoids extracted from its root have important roles in anti-inflammatory [28], anti-oxidation [29], liver protection [30], anti-tumor [31], etc. There are increasing evidences that clinically valuable traits of *I. stachyodes* roots benefit from flavonoids, but the PA accumulation and biosynthesis in *I. stachyoides* roots is still unknown.

**Fig. 1:**
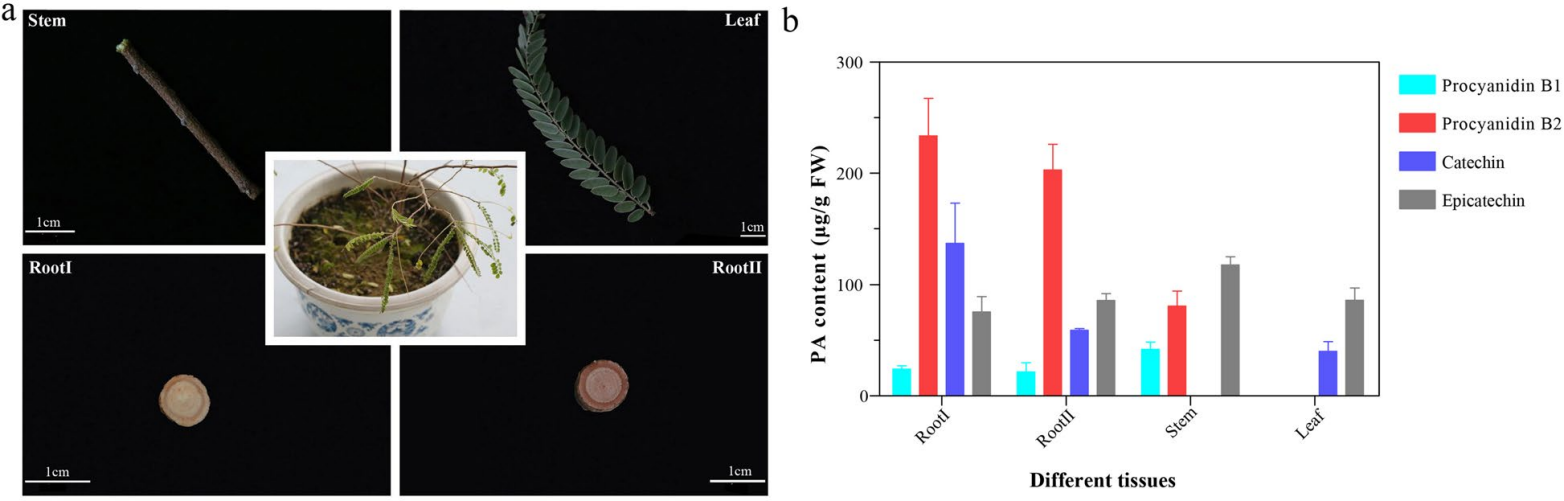
Four tissues morphology and PA content at Leaf, Stem, RootI, and RootII. **a** The morphology of the four tissues. **b** The changes of PA content at four tissues. Each value represents a mean ± SD of three independent biological replicates

Currently, the regulation mechanism of PA synthesis at the gene level by transcriptome analysis has been deeply studied in other plants, such as *persimmon *[32], *Malus Crabapple *[33], *Brassica napus *[34], *pinto bean *[35], *cranberry beans *[36]. However, there is still a lack of genomic data on the regulatory mechanism of PA biosynthesis in *I. stachyodes* roots, which has affected the breeding process of red root varieties. In this study, we performed RNA-seq analysis on *I. stachyodes* in different tissues (Fig. 1a) to identify candidate regulators of PA accumulation. Furthermore, we conducted a TGMI network analysis to investigate PA biosynthesis pathway‑specific regulators involved in *I. stachyodes* roots. The results of our research can provide help for the study of the PA biosynthesis mechanism of *I. stachyodes* roots.

## Results

### Quantitation of PA content in four different tissues

Figure 1a shows the morphological features of the four tissues Leaf, Stem, RootI, and RootII at the same period. From RootI to RootII, the color of root bark kept rising. RoootI and RootII showed distinct PA accumulation patterns compared to Stem and Leaf (Fig. 1b). Levels of procyanidin B2 differed significantly between ground parts (leaves, stems) and underground parts (roots). Procyanidin B2 levels for RootII (208.4 µg/g FW) and RootI (233.7 µg/g FW) were high and there is no significant difference between the two (Additional file 1: Table S1). The content of another polymeric procyanidin B1 in roots is not higher than that in stems. But due to the proportion of the bark in the sample taken in RootI was significantly more than that in RootII, thus the content of proanthocyanidins in RootII was slightly lower than that in RootI.

### Transcriptome sequencing and de novo assembly of *I. stachyodes*

 In order to fully construct the transcriptome of *I. stachyodes*, four major tissues, including Leaf, Stem, RootI, and RootII, were sampled for RNA isolation. Distinct cDNA libraries of those tissues were constructed and sequenced, resulting in a total of 102 G raw reads. After the removal of adapters, poly-N-containing reads, and low-quality sequences from the raw data, approximately 98 G clean reads were obtained with 98.36% Q20 and 94.89% Q30 bases, and the clean data of each sample is above 7.18 G. The GC percentage in ground parts (leaves, stems) and underground parts (roots) were an average of 44.6% and 44.65%, respectively (Additional file 2: Table S2). Trinity assembly program was used for de novo assembly of all sample clean data, after optimizing the assembly results, a set of 110,779 non-redundant unigenes was obtained. The total length of the unigenes was 92,992,355 bp, with an average length of 839.44 bp and the N50 and E90N50 value of 1,540 and 3,117 bp, respectively. In the 110,779 unigenes, 24,442 unigenes (22.1%) were greater than 1 kb in length (Table 1). The size distribution of unigenes is shown in Additional file 3: Figure S1.

**Table 1:** Summary of sequence assembly and function annotation of the *I. stachyodes* transcriptome

Assembly	Values	
Total number of unigenes	110,779	
Total length (bp)	92,992,355	
Average length (bp)	839.44	
N50 (bp)	1,540	
E90N50 (bp)	3,117	
Unigenes (> 1 kb)	24,442	
Unigenes (> 2 kb)	11,713	
**Annotation**		
** Protein database searches**	Values	**Percentage (%)**
BLASTx against NR	57,789	52.17
BLASTx against Swiss-Prot	46,559	42.03
BLASTx against COG	53,295	48.11
BLASTx against Pfam	46,581	42.05
All annotated transcripts	63,863	57.65
**Functional classification and pathway mapping**		
Annotated with Gene Ontology (GO) terms	45,036	40.65
Annotations against KEGG	32,950	29.74

Further, the clean reads of each sample are compared with the Trinity-assembled transcriptome. The average alignment rate was 83.66%, indicating that a high-quality de novo assembled transcriptome was obtained.

### Functional annotation and classification

**Fig. 2:**
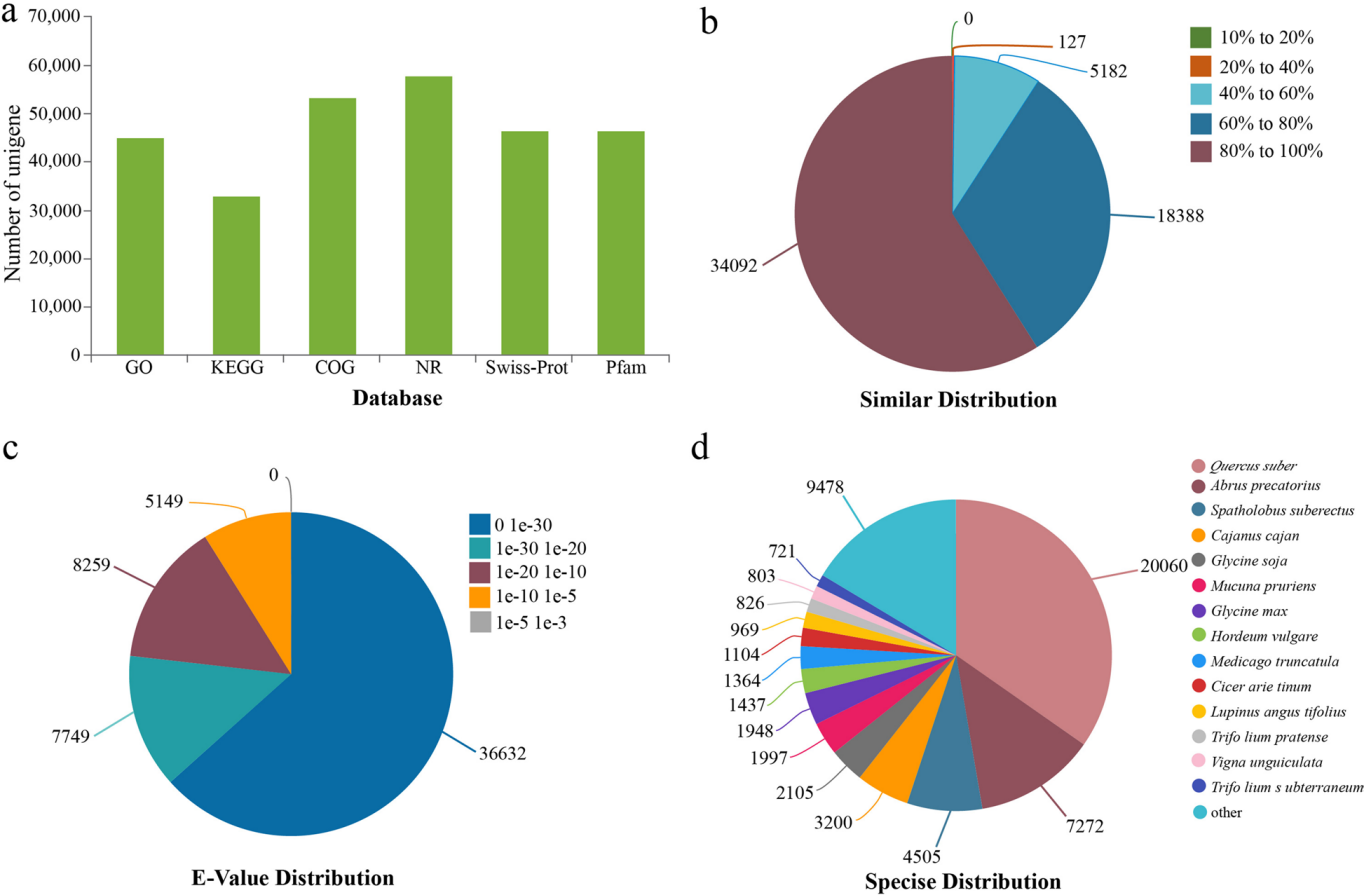
Blast results of the assembled unigenes and the *I. stachyodes* transcriptome homology searches against the NR database. **a** Functional annotation of unigene. **b** Similarity distribution of top BLAST hits for each unigene. (**c**) E-value distribution of BLAST hits with a cut off E-value of 1e-5. **d** Species distribution for top BLAST hits in the Nr database

After assembly, the 110,779 transcriptome sequences were annotated by six databases (NR, Swiss-Prot, Pfam, COG, GO, and KEGG) to obtain similarity sequence and the corresponding annotation information. Gene annotation showed that 63,863 unigenes were successfully annotated in Pfam, Swiss-Prot, NR, COG, KEGG, and GO databases. The number and mapping rates of unigenes against the Pfam, Swissprot, GO, COG, and KEGG databases were 42.05%(46,581)ˎ 42.03%(46,559)ˎ 40.05%(45,036)ˎ 48.11%(53,295)ˎ 29.74%(32,950), respectively (Fig. 2a) (Table 1). 34,092 unigenes had high similarity (greater than 80%) in mapped sequences with Nr database and 36,632 unigenes (63.39%) had significant homology (e-value < 1e-30) (Fig. 2b and 2c). Species distribution analysis showed that only 20,060 unigenes (34.71%) had high homology with the genes from *Quercus suber*, followed by *Abrus precatorius* (7,272, 12.58%), *Spatholobus suberectus* (4,505, 7.8%), while 9,478 unigenes had high homology with sequences from other organisms (Fig. 2d).

The functions of all unigenes were classified by using the Nr annotation and Gene Ontology (GO) classification, and a total of 45,036 unigene gene functions were described under three main divisions (biological process, cellular component, and molecular function) (Additional file 4: Table S3). The predominant group in each of the biological processes, cellular components, and molecular functions was “cellular process” (21,820, 50.13%), “cell part” (22,578, 50.13%), and “binding” (26,213, 26.21%), respectively (Additional file 3: Figure S2). To further understand the biological functions and interactions of transcripts, the unigenes of assembled sequences were assigned by the Kyoto Encyclopedia of Genes and Genomes (KEGG) database. A total of 31,215 unigenes were assigned to 148 KEGG pathways using BLASTx, with an e-value < 1e-5, and were assigned to six main categories. “Translation” had the largest number of unigenes (6,455 unigenes) followed by “Carbohydrate metabolism” (4,069 unigenes), “Energy metabolism” (2,764 unigenes), “Amino acid metabolism” (2,457 unigenes), “Folding, sorting and degradation” (2,036 unigenes), and “Transport and catabolism” (1,853 unigenes) (Additional file 3: Figure S3) (Additional file 5: Table S4).

### Analysis of differentially expressed genes

**Fig. 3:**
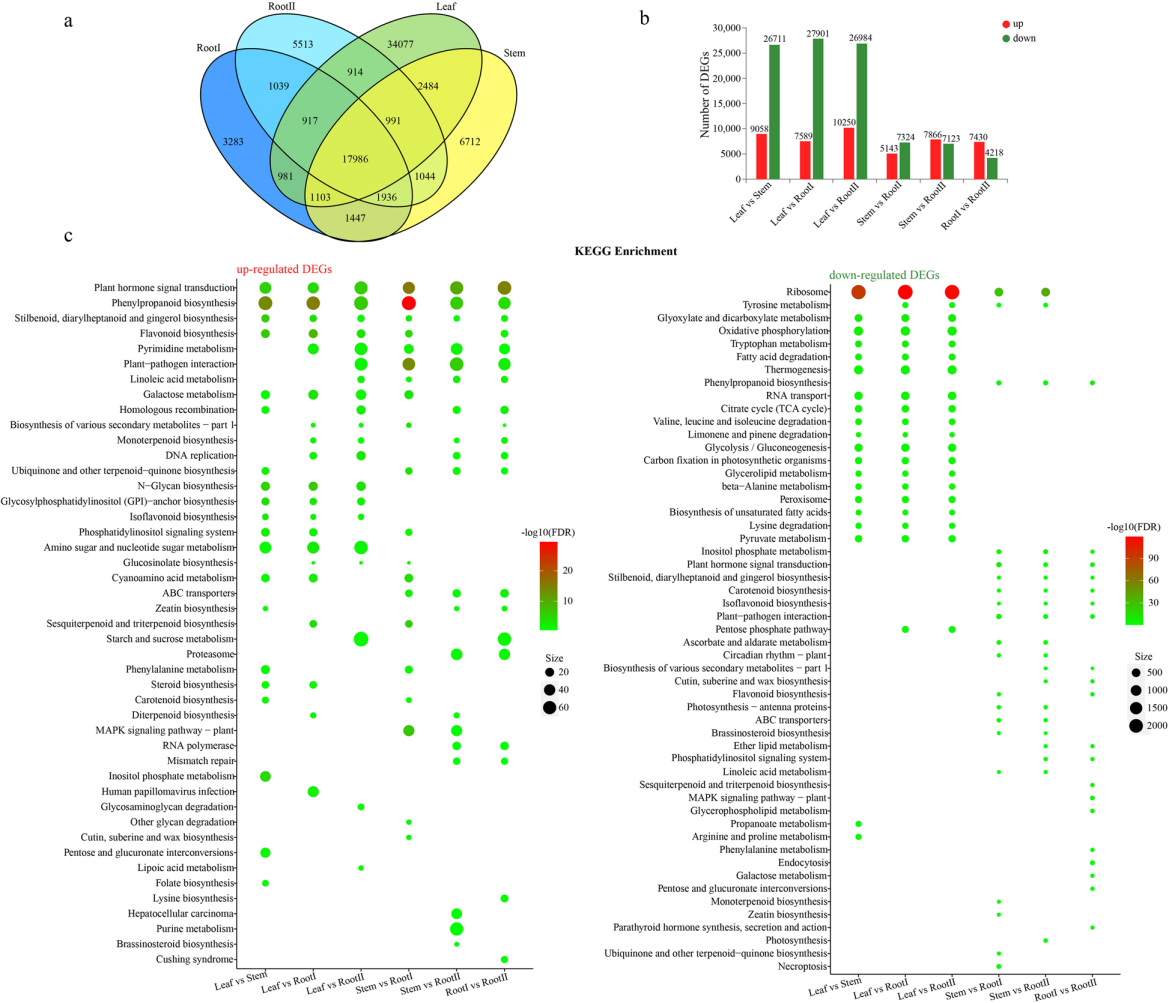
The number and KEGG enrichment of DEGs. **a** Distribution of the unigenes of the four libraries. **b** The red columns indicate the up-regulated DEGs and the green columns represent the down-regulated DEGs in six pair-wise comparisons (FDR ≤ 0.05 and an absolute value of log 2 Ratio ≥ 2 was used as the significant threshold for DEGs). **c** The top 20 enriched KEGG pathways of DEGs. The y axis shows the metabolic pathway terms, and the x axis shows the different comparison groups. The size of the plotted circle indicates the Sample number in this pathway terms. The fill color is scaled to the -log10(FDR). (FDR < 0.05)

The unigenes from different tissues of *I. stachyodes* were compared using assembled data as a reference (Fig. 3a). Under the criteria of *p*-adjust < 0.05 and |log2FC|≥ 2, a total of 11,648 differentially expressed genes (DEGs) between Leaf and Stem were identified. Among them, 9,058 genes were up-regulated, and 26,711 genes were down-regulated. In addition, 35,490 (7,589 up-regulated and 27,901 down-regulated), 37,234 (10,250 up-regulated and 26,984 down-regulated), 12,467 (5,143 regulated and 7,324 down-regulated), 14,989 (7,866 up-regulated and 7,123 down-regulated), 11,648 (7,430 up-regulated and 4,218 down-regulated) were identified in the comparison of Leaf vs RootI, Leaf vs RootII, Stem vs RootI, Stem vs RootII, RootI vs RootII, respectively (Fig. 3b). To obtain a comprehensive understanding of DEGs, gene ontology (GO) and kyoto encyclopedia of genes and genomes (KEGG)-based functional enrichment was conducted. According to GO assignments, a total of 27,999 up-regulated DEGs (Additional file 6: Table S5) and 56,166 down-regulated DEGs (Additional file 6: Table S6) were divided into three main categories: biological process, cellular component, and molecular function. Overall, the up-regulated and down-regulated DEGs in different groups were significantly enriched in the same or different GO terms (Additional file 3: Figure S4). Among the KEGG pathway analysis, biosynthesis of secondary metabolites such as “Phenylpropanoid biosynthesis” and “Flavonoid biosynthesis” represented the top twenty enriched KEGG pathways, especially in the up-regulated DEGs of group Leaf vs Stem, Leaf vs RootI, Stem vs RootI, RootI vs RootII, and the down-regulated DEGs of group Stem vs Root and RootI vs RootII (Additional file 6: Table S7, Table S8). Notably, the down-regulated DEGs in group Leaf vs RootI and Leaf vs RootII were significantly enriched in Ribosome, and only group Stem vs RootI had up-regulated DEGs that significantly enriched in the “Phenylalanine metabolism pathway”, containing 70 upregulated DEGs (Fig. 3c). DEGs between root tissue and other tissues, such as PAL, 4CL, CHS, CHI, C4H, DFR, ANR, ANS, and LAR, were in the significantly enriched pathway terms “Phenylpropanoid biosynthesis”(map00940) and “Flavonoid biosynthesis”(map00941), seemed relevant to the goal of our study (Table 2).

**Table 2:** The situation of the concerned DEGs in the significantly enriched pathway terms

Gene ID	Annotation	Leaf vs Stem	Leaf vs RootI	Leaf vs RootII	Stem vs RootI	Stem vs RootII	RootI vs RootII
*TRINITY_DN13804_c0_g1*	PAL	yes|down	no|down	yes|down	yes|up(map00940)	no|up	no|down
*TRINITY_DN23630_c0_g2*	PAL	yes|up(map00940)	yes|up(map00940)	yes|up(map00940)	no|up	no|down	yes|down(map00940)
*TRINITY_DN5709_c1_g1*	PAL	yes|up(map00940)	yes|up(map00940)	no|down	no|down	yes|down(map00940)	yes|down(map00940)
*TRINITY_DN5784_c0_g1*	PAL	no|down	yes|up(map00940)	no|up	yes|up(map00940)	no|up	yes|down(map00940)
*TRINITY_DN5784_c0_g2*	PAL	yes|down	no|down	yes|down	yes|up(map00940)	no|up	yes|down(map00940)
*TRINITY_DN4766_c0_g1*	C4H	no|up	yes|up(map00941)	yes|up	yes|up	no|up	yes|down
*TRINITY_DN7054_c0_g1*	C4H	no|down	yes|up(map00941)(map00940)	no|up	yes|up(map00941)(map00940)	yes|up(map00940)	yes|down(map00941)(map00940)
*TRINITY_DN11429_c0_g1*	4CL	yes|down	no|up	yes|down	yes|up(map00940)	yes|down(map00940)	yes|down(map00940)
*TRINITY_DN14591_c0_g1*	4CL	yes|down	no|up	no|down	yes|up(map00940)	yes|up(map00940)	yes|down(map00940)
*TRINITY_DN1781_c1_g5*	4CL	yes|up(map00940)	no|up	yes|up(map00940)	no|down	no|down	no|up
*TRINITY_DN2134_c2_g2*	4CL	yes|down	yes|down	yes|down	no|down	yes|down(map00940)	no|down
*TRINITY_DN26629_c1_g1*	4CL	yes|down	yes|down	yes|down	no|up	yes|up(map00940)	yes|up(map00940)
*TRINITY_DN42897_c0_g1*	4CL	no|down	yes|up(map00940)	no|up	yes|up(map00940)	no|up	no|down
*TRINITY_DN4760_c0_g1*	4CL	yes|up(map00940)	yes|up(map00940)	yes|up(map00940)	no|up	no|up	no|down
*TRINITY_DN36039_c0_g1*	CHS	no|down	no|up	no|down	yes|up(map00941)	no|down	yes|down(map00941)
*TRINITY_DN6204_c1_g1*	CHS	no|down	yes|up(map00941)	no|up	yes|up(map00941)	no|up	yes|down(map00941)
*TRINITY_DN7831_c0_g1*	CHS	yes|up(map00941)	yes|down	yes|up(map00941)	yes|down(map00941)	no|down	yes|up(map00941)
*TRINITY_DN7831_c0_g2*	CHS	no|up	yes|down	no|up	yes|down(map00941)	no|down	yes|up(map00941)
*TRINITY_DN8474_c0_g1*	CHS	no|up	yes|up(map00941)	no|down	yes|up(map00941)	no|down	yes|down(map00941)
*TRINITY_DN8489_c0_g1*	CHS	no|up	yes|up(map00941)	no|up	yes|up(map00941)	no|up	yes|down(map00941)
*TRINITY_DN2469_c0_g1*	CHI	yes|up(map00941)	yes|up(map00941)	yes|up(map00941)	no|up	no|up	no|down
*TRINITY_DN5151_c0_g1*	CHI	no|up	no|up	no|down	no|down	yes|down	yes|down(map00941)
*TRINITY_DN50705_c0_g1*	F3H	yes|up(map00941)	yes|up(map00941)	yes|up(map00941)	no|down	no|down	no|up
*TRINITY_DN9849_c0_g3*	F3′H	yes|up(map00941)	no|up	yes|up(map00941)	no|down	no|down	no|up
*TRINITY_DN1954_c0_g1*	DFR	yes|up(map00941)	yes|up(map00941)	yes|up(map00941)	yes|down(map00941)	no|down	no|up
*TRINITY_DN1954_c0_g2*	DFR	no|down	yes|down	yes|down	yes|down(map00941)	no|down	yes|up(map00941)
*TRINITY_DN18473_c0_g1*	ANS	no|down	yes|down	no|down	yes|down(map00941)	no|up	yes|up(map00941)
*TRINITY_DN1277_c0_g1*	ANR	yes|up(map00941)	yes|up(map00941)	yes|up(map00941)	no|down	no|down	no|down
*TRINITY_DN10135_c0_g3*	LAR	yes|up(map00941)	no|down	yes|up(map00941)	yes|down(map00941)	yes|down	yes|up(map00941)
*TRINITY_DN26566_c0_g1*	LAR	yes|down	no|up	no|up	yes|up(map00941)	yes|up	no|up

Phenylpropanoid biosynthesis (map00940); Flavonoid biosynthesis (map00941)

### Expression patterns of PA biosynthesis potential pathway structural genes in different tissues

**Fig. 4:**
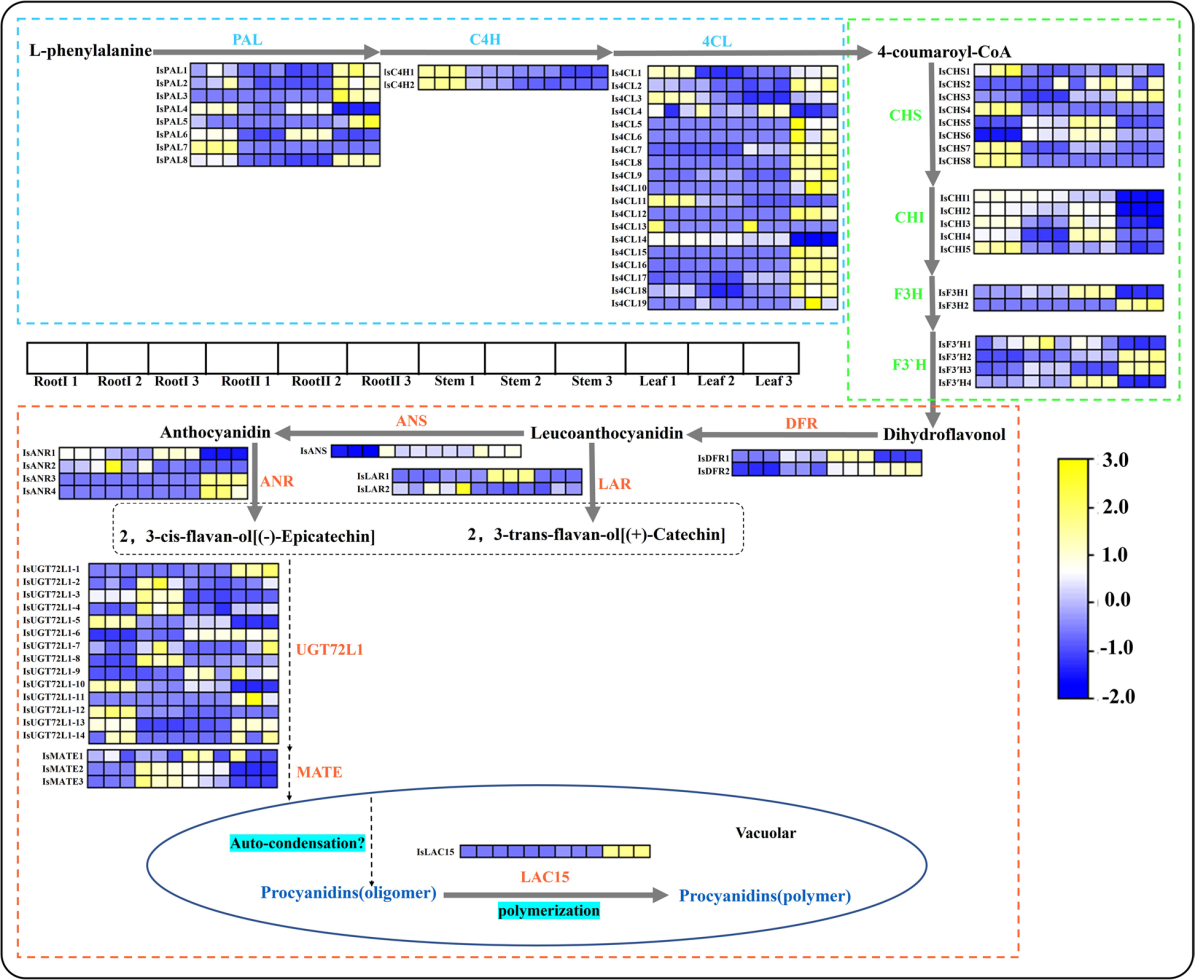
An overview of PA biosynthesis pathway and PA-related structural genes expression across *I. stachyodes* organ type. Abbreviations are as follows: PAL (Phenylalanine ammonia lyase), C4H (cinnamate 4-hydroxylase), 4CL (4-coumaroyl CoA ligase), CHS (chalcone synthase), CHI (chalcone isomerase), F3H (flavanone 3-hydroxylase), F3′H (flavonoid 3′-monooxygenase), DFR (dihydroflavonol-4-reductase), LAR (leucoanthocyanidin reductase), ANS (anthocyanidin synthase), ANR (anthocyanidin reductase), MATE (multidrug detoxification and extrusion), UGT72L1 (UDP-glycosyltransferase), and LAC15 (laccase 15). yellow is high expression, blue is low expression

A large number of PAs were detected in *I. stachyodes* roots, and the two-year-old *I. stachyodes* root with red color is usually used as the medicinal harvesting standard, which means the content of flavonoids in the root of two-year-old *I. stachyodes* is higher [27]. To further investigate these important findings, the transcriptome of different tissues were compared to dig out the key genes in the metabolism of red root related to the growth years of *I. stachyodes*. In total, 75 unigenes that encoded 14 enzymes in the flavonoid and PA biosynthesis pathways were identified using BLASTp with previously identified *Arabidopsis thaliana* genes annotated in KEGG and additional literatures [37–39] (Additional file 7: Table S9). The normalized expression profiles of all the putative PA biosynthesis unigenes found in the *I. stachyodes* transcriptome were shown in Fig. 4. The biosynthesis pathway structural genes of PAs have been mainly divided into three parts [34]. The general biosynthetic genes (GBGs) including *PAL*, *C4H,* and *4CL* are marked in blue, the EBGs including *CHS*, *CHI*, *F3H*, and *F3′H* are marked in green, while the LBGs including *DFR*, *ANS*, *ANR*, *LAR*, *UGT72L1*, *MATE* and *LAC15* are marked in red.

In general, most of the pathway structural genes had significantly tissue-specific expression, which must be related to the accumulation level of PAs in various tissues. *IsUGT72L1-2*, *IsUGT72L1-3*, *IsUGT72L1-4*, *IsUGT72L1-7*, *IsUGT72L1-8*, *IsANR2* had the same expression pattern with *IsLAR2* (*TRINITY_DN26566_c0_g1*), showed specific high expression in RootII tissue. In the result of correlation between the expression of pathway structural genes and the measured PA content, the correlation with procyanidin B2 is *IsLAR2*(0.479), *IsANR2*(0.654), *IsUGT72L1-3*(0.84), sequentially show a strong positive correlation (Additional file 3: Figure S5). Similarly, *IsF3′H1*ˎ *IsMATE2*ˎ *IsMATE3* showed highly up-regulated expression in RootII tissue, and had a strong positive correlation with the accumulation level of procyanidin B2 and B1. Interestingly, *IsCHS6*, *IsANS*, *IsUGT72L1-4*, *IsDFR2* showed specific low expression in RootI tissue, the correlation with epicatechin is *IsANS*(0.371), *IsDFR2*(0.509), *IsCHS6*(0.739) sequentially show a strong positive correlation. *IsCHS1*, *IsCHS4*, *IsCHS7*, *IsCHS8*, *IsCHI5*, *IsC4H1,* *IsC4H2*, *IsUGT72L1-12*, *Is4CL11*, *IsPAL7* showed specific high expression in RootI tissue, and showed highly strong positive correlation with catechin and procyanidin B2 (Additional file 3: Figure S5). From the results of our association analysis, it can be seen that the synthesis of PA synthesis precursors is mainly regulated in RootI, while the synthesis of procyanidin B2 is critically regulated in RootII, and genes involved in this process may play a key regulatory role.

### Identification of regulators of PA biosynthesis genes in *I. stachyodes* roots

**Fig. 5:**
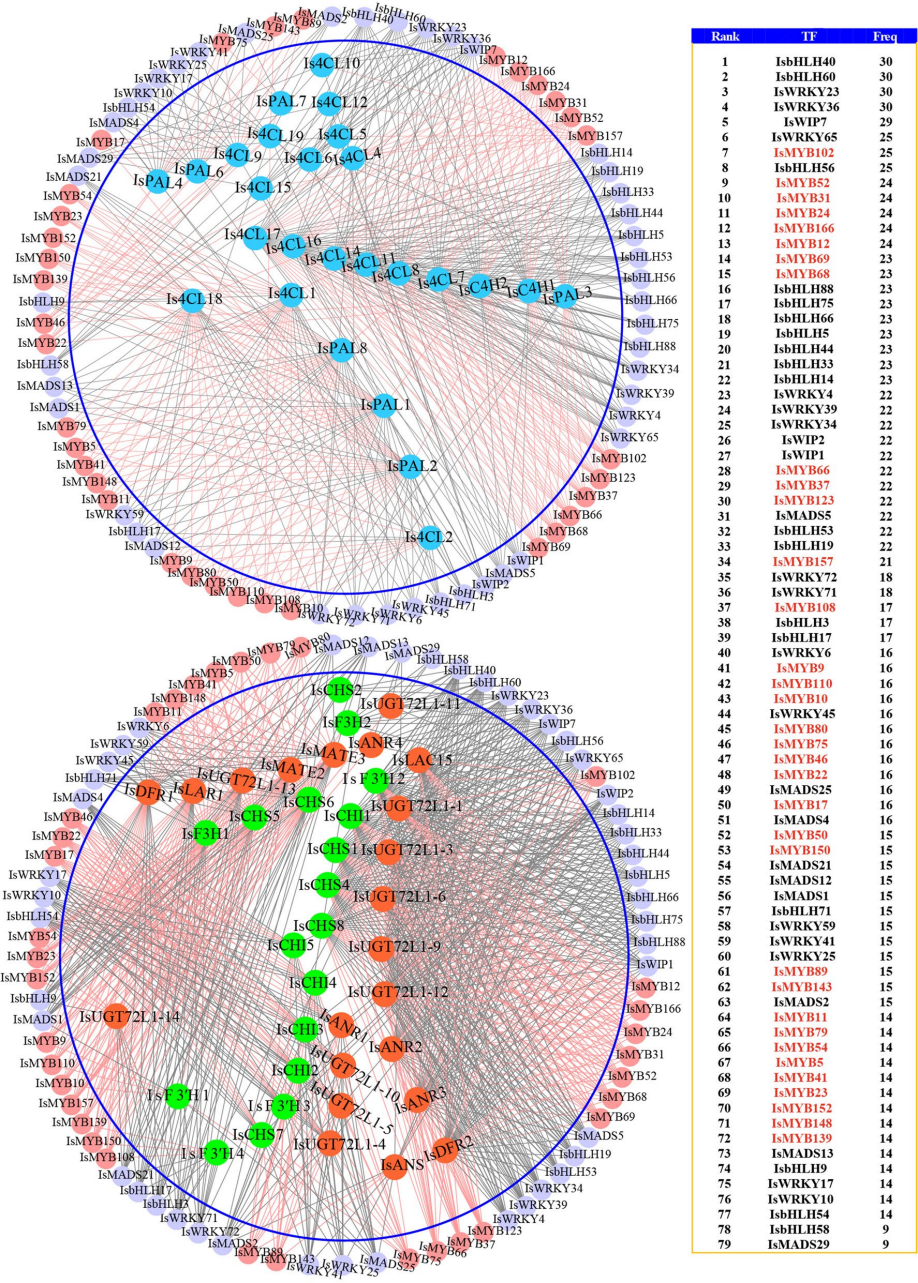
Network analysis of the most positive known PA biosynthesis pathway regulators MYB, bHLH, WRKY, MADS, and WIP. Regulatory network of GBGs (top) and regulatory network of EBGs and LBGs (bottom) generated by TGMI algorithm for the *I. stachyodes* PA biosynthesis pathway using the high-throughput data yielded from treatment versus control. Blue nodes represent GBGs. Green nodes represent EBGs. Orange-red nodes represent LBGs. All other nodes are TFs regardless of what colors they are. Light coral nodes represent the most positive known pathway regulators MYB

PA biosynthesis is controlled by regulatory networks that consist of TFs or regulatory complexes in different species [13, 40]. In order to comprehensively reveal the regulatory network of PA biosynthesis, the expression data of the PA pathway genes and all the TFs were extracted from *I. stachyodes* transcriptome dataset (Additional file 7: Table S9; Additional file 8: Table S10), and were applied to co-expression analysis using the TGMI algorithm. The triple gene blocks were identified by the TGMI algorithm with a cut-off significance level of 0.05 (Additional file 9: Table S11). The interference frequencies of TFs on pathway genes were displayed in descending order (Additional file 10: Table S12). Among the top 185 TFs regulators, which interfere with the pathway genes with the highest frequencies, in the lists identified by TGMI, 34 IsMYBs, 18 IsbHLHs, 15 IsWRKYs, 9 IsMADSs, 3 IsWIPs are known PA pathway regulators supported by literature. These TFs were further combined to generate a circular network, as shown in Fig. 5. It is perceivable that the core pathway regulator MYB highlighted in a light coral color, is considered to be a top candidate that plays a central role in the expression regulation of pathway structural genes [13, 14].

**Fig. 6:**
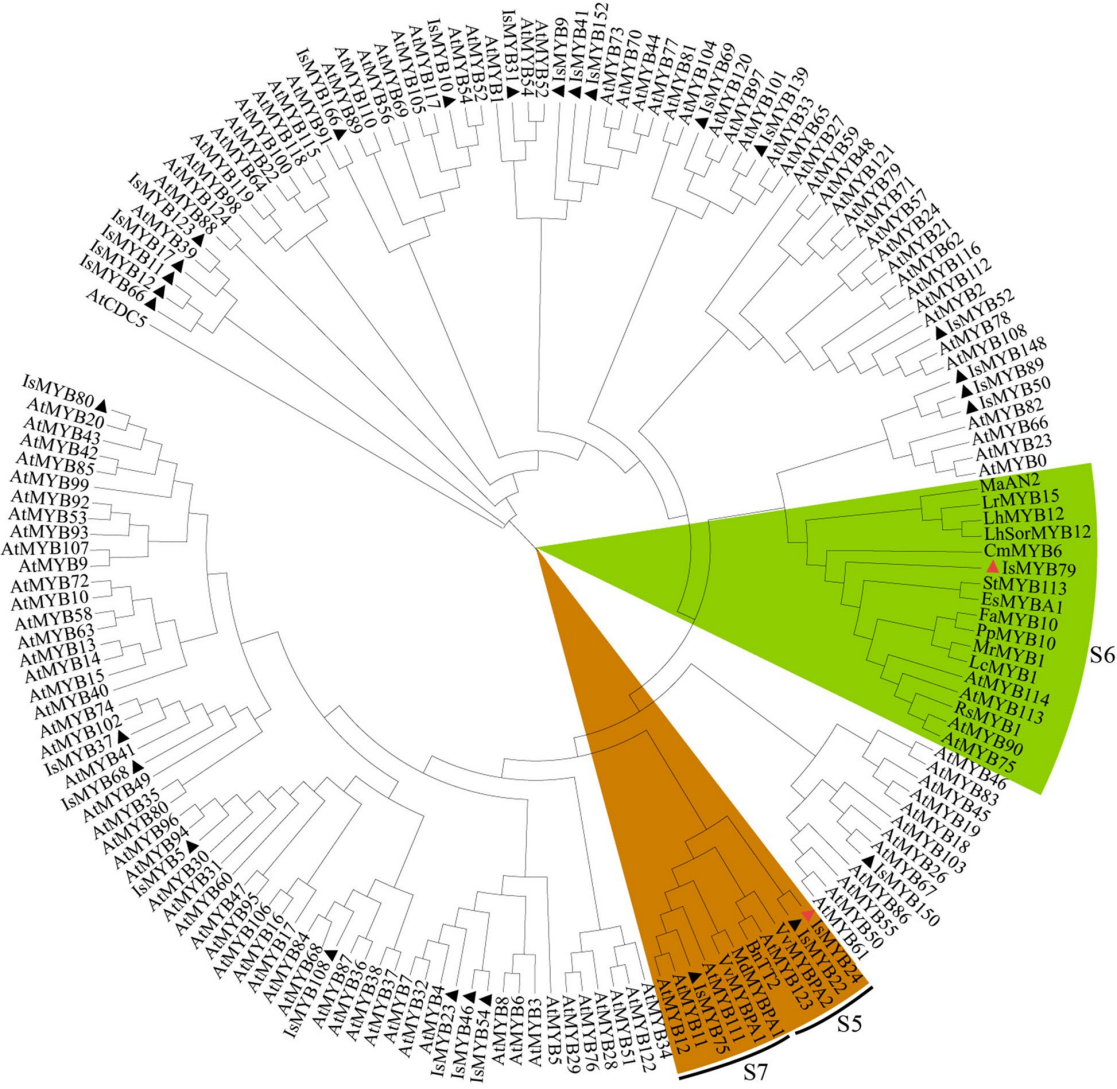
Phylogenetic analyses of the combinatorial TFs MYB. Phylogenetic tree constructed with MYBs of *Arabidopsis thaliana* and proteins related to flavonoids biosynthesis in other species, including *Vitis vinifera* VvMYBPA1 (NP_001268160.1), *Raphanus sativus* RsMYB1 (AKM95888.1), *Camellia sinensis* CsMYB2 (AEI83426.1), *Brassica napus* BnTT2 (ABI13035.1), *Lilium hybrid* division VII LhSorMYB12(BAJ22983.1), *Lilium hybrid* division I LhMYB12(BAO04194.1), *Lilium regale* LrMYB15(BAU29930.1), *Prunus persica* PpMYB10 (ADK73605.1), *Litchi chinensis* LcMYB1 (APP94121.1), *Chrysanthemum x morifolium* CmMYB6 (QUP79395.1), *Fragaria x ananassa* FaMYB10 (QIZ03070.1), *Morella rubra* MrMYB1 (ADG21957.1), *Epimedium sagittatum* EsMYBA1 (AGT39060.1), *Euproctus montanus* EsAN2 (AFY04089.1), *Solanum tuberosum* StMYB113 (AND01219.1), *Muscari armeniacum* MaAN2 (ASF20090.1), *Vitis vinifera* VvMYBPA2 (NP_001267953.1), *Malus domestica* MdMYBPA1(NP_001315766.1). IsMYBs protein sequences screened from the TGMI algorithm are labelled with triangles. Proteins labelled with red triangles belong to the clades of proanthocyanidin synthesis. The tree was constructed with the NJ method (1000 replications of bootstrap test) using the MEGAX program

**Fig. 7:**
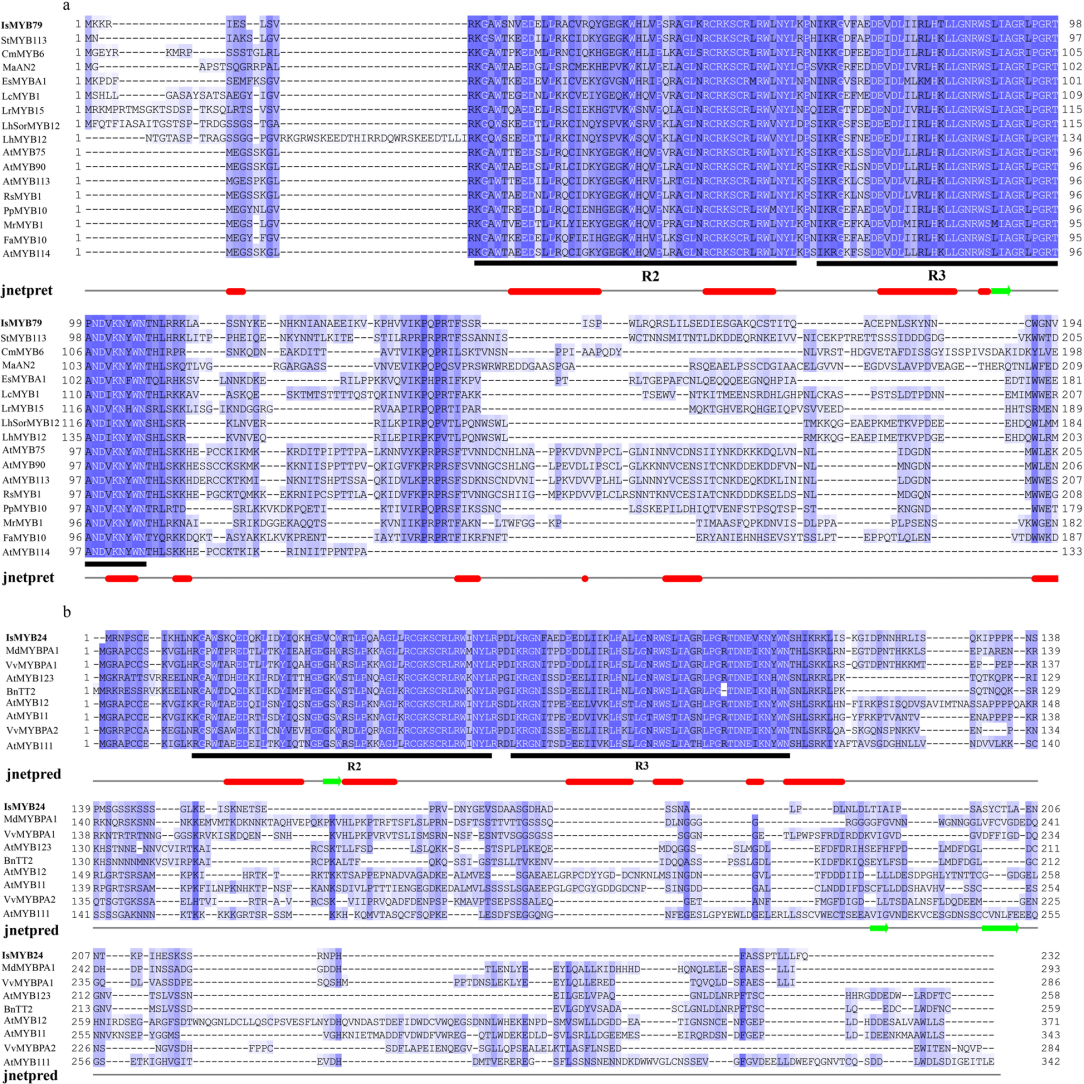
Multiple alignments analyses of key MYB TFs. **a** Multiple alignments of IsMYB79 and IsMYB24 **b** amino acid sequences and other published flavonoid-related MYBs. Black lines indicate R2 and R3 domain in MYB family. Jnetpred means secondary structure prediction results of IsMYB79 and IsMYB24 proteins, red indicates tubes, and green arrows indicate sheets

In brief, co-expression analysis identified numerous potential interactive regulators of PA biosynthesis, involving 34 IsMYBs, 18 IsbHLHs, 15 IsWRKYs, 9 IsMADSs, and 3 IsWIPs (Additional file 11: Table S13) were chosen for heatmap analysis (Additional file 3: Figure S6). It is worth noting that *IsWRKY45*, *IsMYB24*, *IsbHLH33*, *IsMYB80*, *IsMYB9*, *IsMYB52*, *IsMADS12*, *IsMYB68*, *IsbHLH14*, *IsMYB79*, *IsMYB69*, *IsMYB23* exhibited a higher expression level in RootII. Genes with same or similar expression patterns are often under the regulation of the same molecular mechanism [41]. Thus, we should focus on the network in RootII in order to further dig out key regulators affecting the synthesis of PAs in roots. Two mainly different subnetworks were detected. In sub-network one, four MYBs (*IsMYB23*, *IsMYB79*, *IsMYB9*, *IsMYB80*), one *IsWRKY45*, one *IsMADS12*, and 2 LBGs (*IsMATE2*, *IsMATE3*) were co-expressed. Meanwhile, in sub-network two, four MYBs (*IsMYB24*, *IsMYB52*, *IsMYB68*, *IsMYB69*), two bHLHs (*IsbHLH14*, *IsbHLH33*), and only with 1 LBGs *IsANR2* co-expressed. These TFs also have a strong correlation with the level of PAs in *I. stachyodes* (Additional file 3: Figure S7). R2R3-MYB generally plays a central role in regulating target genes in PA pathways [13, 14]. To further screen out the PA-related R2R3-MYB proteins and predict their functions, we constructed a phylogenetic tree comprising the 34 IsMYBs proteins along with 126 *Arabidopsis* R2R3-MYB proteins and 16 proteins related to this process in other plant species (Fig. 6). *IsMYB79* with higher expression level in RootII than other tissues, was clustered in subgroup 6 of the MYB gene family, such as *AtMYB90*, *AtMYB75*, *AtMYB114*, and *AtMYB113* in *Arabidopsis thaliana *[42]. The overexpression of *AtMYB75* or *AtMYB90* in purple transgenic tobacco plants strongly enhances anthocyanin contents via upregulating all of the anthocyanin biosynthetic genes [43]. *IsMYB24*, another higher expressed in RootII, which was clustered in subgroup 5 of the MYB gene family, *AtMYB123*, and TT2-type genes were involved in anthocyanin and PA biosynthesis regulation [13, 44]. *IsMYB75* clustered in subgroup 7 and *IsMYB22* clustered in subgroup 5, but they specifically expressed in leaf tissues not in roots (Fig. 6). To determine the characterization of *IsMYB24* and *IsMYB79*, homologous sequence alignment was carried out using deduced amino acid sequences and other published flavonoid-related genes amino acid sequences (Fig. 7). The results show that *IsMYB79* and *IsMYB24* have the general characteristics of R2R3-MYB gene family, and contained R2 and R3 domains. *IsMYB79* was closely related to other published anthocyanin-related MYBs, which were promoting pigmentation, such as *CmMYB6 *[45], *MaAN2 *[46], *LrMYB15 *[47], *StMYB113 *[48], *PpMYB10 *[49], *MrMYB1 *[50] had been studied in model plant tobacco, the molecular mechanism of regulating anthocyanin accumulation has been basically clarified. EsMYBA1 influences pigmentation in the leaves, flowers, and flower buds [51]. *LhMYB12* and *LhSorMYB12* in the *Lilium* species control anthocyanin pigmentation in whole tepals [52]. The transcriptional activation of *RsMYB1 *[53] resulted the anthocyanin pigmentation. The highly homology indicated that the function of these MYBs was similar. In addition, *IsMYB24* was closely related to *VvMYBPA2*, which plays crucial roles in regulating PA biosynthesis [54]. Therefore, *IsMYB79* and *IsMYB24* were similar to other flavonoid-related genes, which may play an important role in promoting root pigmentation.

## Discussion

### PA compounds in *I. stachyodes* roots

The result of our study showed that procyanidin B2 is the most important flavonoid in *I. stachyodes* roots, composed of two molecules of epicatechin [55]. Therefore, in *I. stachyodes* roots, PAs maybe are primarily epicatechin-based, similar to the situation in seed coats of the model plants *Arabidopsis thaliana* and *Medicago truncatula* [56]. Moreover, Cyanidin (Cy) was the main coloration anthocyanin component in *I. stachyodes* roots. Similarly, in apple, one of the most common anthocyanin pigments is cyanidin, which, in the form of cyanidin 3-O-galactoside, is the pigment chiefly responsible for red skin coloration [57]. In this regard, Cy appears to be the main anthocyanins determining the red color of *I. stachyodesin* roots. Once formed, the unstable Cy would be converted to the colorless epicatechin, which would eventually form procyanidin B2 via later glycosylation and other reactions.

### The PA biosynthesis pathway in *I. stachyodes* roots

The KEGG database revealed that upregulated DEGs were significantly enriched in “Phenylpropanoid biosynthesis”, which provided a precursor for the biosynthesis of flavonoids including flavonol, anthocyanidin, and PA [58]. In addition, 75 DEGs correlated with PA biosynthesis were identified and found to encode PAL, C4H, 4CL, CHS, CHI, F3H, F3′H, DFR, ANS, ANR, LAR, UGT72L1, MATE, and LAC15. Of these, *IsF3′H1*, as a key rate-limiting enzyme in the process of flavonoids biosynthesis [59], showed a higher expression level at RootII compared with other tissues. The high expression of genes encoding F3′H would catalyze and synthesize a large amount of dihydroquercetin. ANR enzyme first catalyzes anthocyanins to generate flav-enol intermediates, then ANR enzyme catalyzes flav-en-ol intermediates to generate flavan-3-ol or flavan-3-ol carbocation, which participates in the subsequent transport and polymerization of PA [60]. In this study, we found that *IsANR2* was up-regulated in RootII, leading to the accumulation of (-)-Epicatechin. *IsLAR2* is also found up-regulated in RootII, which can not only convert anthocyanins into ( +)-catechins, but also convert 4β-(S-cysteinyl)-epicatechin back to epicatechin, the starter unit in PAs, thereby regulating the relative proportions of starter and extension units and consequently the degree of PA oligomerization [4].

The synthesis of dimeric flavan-3-ols (procyanidin B2) is the key metabolic pathway of PAs synthesis in *I. stachyodes* roots. Studies have reported that procyanidin B2 is produced by the polymerization of (–)-epicatechin carbocation and (–)-epicatechin [4]. Glycosylation was the precondition for flavonoids to be transported from endoplasmic reticulum to vacuoles, and epicatechin glycoside was the potential precursor of PA polymerization [61]. UGT72L1 can catalyze the glycosylation of epicatechin to produce epicatechin glycoside [10]. In this study, we found *IsUGT72L1-3* up-regulated in RootII, which has a great connection with the transport process of PA synthesis in *I. stachyodes* roots. Two genes (*IsMATE2*, *IsMATE3*) code MATE also found up-regulated in RootII in our study, can preferentially transport epicatechin-3′-O-glucoside across membranes in yeast assay systems [11]. So far, the only known enzyme involved in PA oxidation and polymerization in *Arabidopsis thaliana* is TT10 (LAC15), and other enzymes involved in polymerization and oxidation still need to be identified. The result of this study found LAC15 not expressed in the root tissue, thus we deduced that the polymerization of PAs maybe not the key step in *I. stachyodes* roots.

### Identification of PA biosynthesis key genes and TFs in *I. stachyodes* root

TGMI has been used to study lignin biosynthesis pathway in *Arabidopsis thaliana *[62], *Populus *[63], and *Populus trichocarpa *[64], for identifying which regulatory genes potentially control wood formation. In this study, we also applied the TGMI algorithm to true pathway regulators of PA biosynthesis in *I. stachyodes* roots based on the tissue-specific *I. stachyodes* gene expression datasets. As anticipated, our study identified 34 IsMYBs, 18 IsbHLHs, 15 IsWRKYs, 9 IsMADSs, and 3 IsWIPs regulators that potentially regulate PA biosynthesis in *I. stachyodes* and ranked them to the top of candidate regulatory gene lists (Additional file 3: Figure S6). *IsMYB24*, a homologous gene of PA-related MYB genes in subgroup 5, showed strong correlation with PA biosynthetic genes *IsANR2*. In many plant species, TT2 (AtMYB123) and its homologs are direct activators of genes encoding ANR, LAR, and other enzymes in the PA biosynthesis pathway [13]. Furthermore, TT2 forms a ternary complex with TT8 (bHLH) and TTG1 (WD40) to activate genes related to PA biosynthesis [65]. Similarly, in our study, IsMYB24 may form transcriptional complexes with IsbHLH (IsbHLH14, IsbHLH33), co-expressed with *IsANR2*, to regulate PA biological processes. In addition, *IsMYB79* clustered in subgroup 6 could regulate the expression of LBGs (*IsMATE2*, *IsMATE3*) and biosynthesis of late anthocyanins [14, 66]. As our result, IsMATEs (*IsMATE2*, *IsMATE3*) was also co-expressed with the RootII-specific expression TFs (IsMYB79, IsWRKY45, IsMADS12). MdWRKY11 can increase the expression of *F3H*, *FLS*, *DFR*, *ANS*, and *UFGT* to promote anthocyanin accumulation in apples [67], binds to W-box cis elements in MdMYB10, MdMYB11 and MdUFGT promoters [68]. Thus, we speculate IsWRKY45 could bind to IsMYB79 to affect the synthesis of PAs. But for IsMADS12, there is still no research showing interactive relationship between MYB and MADS.

In summary, MYB TF is the core member of transcriptional complex, and overexpression of transgenic MYB alone will obviously promote PA biosynthesis [69, 70]. Our TGMI algorithm analysis, hierarchical clustering, and PA-related MYB evolutionary trees together determined two important TFs IsMYB24 and IsMYB79. However, the mechanism of action for them is not yet clear and needs further research.

## Conclusions

In this study, the complete transcriptome of *I. stachyodes* was de novo-assembled and annotated for the first time, generating a total of 110,779 non-redundant unigenes, of which 63,863 could be functionally annotated. The high content of procyanidin B2 in *I. stachyodes* roots was associated with up-regulated genes involved in the early and late steps of PA biosynthesis (*F3′H*, *ANR*, *LAR*, *UGT72L1,* and *MATE*), which produce the dihydroquercetin, (-)-Epicatechin, ( +)-catechins, and epicatechin-3′-O-glucoside, ultimately yield procyanidin B2 during these steps of PA synthesis. Simultaneously, *IsANR2* might be regulated by IsMYB24, while IsMATE (*IsMATE2*, *IsMATE3*) could be regulated by IsMYB79. These results may enable further metabolomic and gene functional study in *I. stachyodes*.

## Methods

### Sample preparation and RNA extraction

*I. stachyodes *was grown in Dechangxiang *I. stachyodes* Planting Base in Xiuwen County, Guizhou. The collection date of four tissues (Leaf, Stem, RootI, and RootII) in the year 2019 is 21th, June. And each sample was composed of three biological replicates. The total RNA was extracted from tissue samples using TRIzol® Reagent (Plant RNA Purification Reagent for plant tissue) according the manufacturer’s instructions (Invitrogen, Carlsbard, CA, USA) and genomic DNA was removed using DNaseI (TaKara). Then the integrity and purity of the total RNA quality were determined by 2100 Bioanalyser (Agilent Technologies, Inc., SantaClaraCA, USA) and quantified using the ND-2000 (NanoDrop Thermo Scientific, Wilmington, DE, USA). Only high-quality RNA sample (OD260/280 = 1.8 ~ 2.2, OD260/230 ≥ 2.0, RIN ≥ 8.0,28S:18S ≥ 1.0, > 1 μg) was used to construct sequencing library.

### Measurement of PAs

A 1-g tissue of each sample was finely grinded into powder for PAs extraction. The PAs were extracted in 40% Ethanol solution at 50℃, then the Continued filtrate was drained under 60 °C water bath. The residue was reconstituted, filtered through a 0.22 microporous membrane, and loaded for analysis.

### cDNA library preparation and transcriptome sequencing

The construction of cDNA library and RNAseq was performed by Shanghai Majorbio Bio-Isarm Technology Co., Ltd. (Shanghai, China). Firstly, mRNA was purified from 12 µg of total RNA from four tissues (Leaf, Stem, RootI, and RootII) by using Oligo(dT) magnetic beads, respectively. Then, the mRNA samples were randomly broken into 300 bp fragments and added with fragmentation buffer. The first-strand cDNA was formed via reverse transcription using reverse transcriptase and random hexamer primer using mRNA as a template. Then, second-strand cDNA was synthesized, forming a stable double-stranded structure. These cDNA fragments were ligated with the Illumina paired-end sequencing adaptors. Finally, these libraries were sequenced on a paired-end flow cell using Illumina Novaseq 6000 platform. We obtained 7.18 G of reads from each sample for de novo assembly.

### De novo assembly and Gene annotation

The raw paired-end reads were trimmed and quality controlled by SeqPrep (https://github.com/jstjohn/SeqPrep) and Sickle (https://github.com/najoshi/sickle) with default parameters. Then clean data from the samples (RootI-1, RootI-2, RootI-3, RootII-1, RootII-2, RootII-3, Stem-1, Stem-2, Stem-3, Leaf-1, Leaf-2, Leaf-3) were used to do de novo assembly with Trinity ([71]. Then, the assembly results were filtered by using TransRate software (http://hibberdlab.com/transrate/) and CD-HIT software (http://weizhongli-lab.org/cd-hit/). Finally, the results of optimized assembly were evaluated by using BUSCO (Benchmarking Universal Single-Copy Orthologs, http://busco.ezlab.org) [72]. Annotation of the assembled unigenes was conducted using BLASTX [73] searches against the KEGG, Pfam, Swissprot, and non-redundant (NR) databases, with the public database (E < 1e-5). The gene ontology (GO) annotation information of these unigenes was obtained from the NCBI Nr database by using the program Blast2GO and contains molecular functions, biological processes, and cellular components [74]. Furthermore, the program WEGO [75] classified all unigenes based on the GO annotation information.

### Analysis of DEGs

To identify DEGs between two different samples, the expression level of each transcript was calculated according to the transcripts per million reads (TPM) method. RSEM (http://deweylab.biostat.wisc.edu/rsem/) was used to quantify gene abundances. Essentially, differential expression analysis was performed using the DESeq2/DEGseq/EdgeR with Q value ≤ 0.05, DEGs with |log2FC|> 1 and Q value ≤  0.05(DESeq2 or EdgeR) /Q value ≤  0.001(DEGseq) were considered to be significantly different expressed genes. In addition, functional-enrichment analyses including GO and KEGG were performed to identify which DEGs were significantly enriched in GO terms and metabolic pathways at Bonferroni-corrected P-value ≤ 0.05 compared with the whole-transcriptome background. GO functional enrichment and KEGG pathway analysis were carried out by Goatools (https://github.com/tanghaibao/Goatools) and KOBAS (http://kobas.cbi.pku.edu.cn/home.do).

### Co-expression network analysis and Network visualization

Co-expression networks were generated using the R package Triple Gene Mutual Interaction (TGMI) [62]. Pathway genes were first evaluated by conditional mutual information plus a novel mutual interaction measure (MIM) we discovered. This MIM reflects the regulatory strength exerted by the TF on two pathway genes in the triple gene block. The larger the MIM, the more significant the TF controls two pathway genes. In order to meet the criteria for TGMI, a cut-off significance level of 0.05 was used in the calculation. This resulted in a final network of 183 nodes (genes and TFs) connected by 1,357 edges (str values). Cytoscape (v 3.8.2) [76] was used to visualize the resulting network using the Allegro Layout plugin with an edge-weighted Allegro Fruchterman-Reingold layout algorithm.

### Homolog search, gene identification, and distance analysis

The coding sequence of AtR2R3-MYB was acquired from the TAIR (http://www.arabidopsis.org/) databases. The amino acids of the MYB proteins were used to perform Phylogenetic analysis using MEGAX software with the neighbour-joining statistical method and 1000 bootstrap replicates.

## Supplementary Information

**Additional file 1.** 

**Additional file 2.** 

**Additional file 3.** 

**Additional file 4.** 

**Additional file 5.** 

**Additional file 6.** 

**Additional file 7.** 

**Additional file 8.** 

**Additional file 9.** 

**Additional file 10.** 

**Additional file 11.** 

## Data Availability

The RNA sequencing reads are available in the Sequence Read Archive database of NCBI (BioProject ID: PRJNA817883).

## Abbreviations

PAs: Proanthocyanidins
TFs: Transcription factors
F3′H: Flavonoid 3′ hydroxylase
ANS/LDOX: Anthocyanidin synthase/Leucoanthocyanidin dioxygenase
ANR: Anthocyanidin reductase
LAR: Leucoanthocyanidin reductase
GO: Gene Ontology
KEGG: Kyoto Encyclopedia of Genes and Genomes
MATE: Multidrug detoxification and extrusion
DEG: Differentially Expressed Gene
GBGs: General biosynthetic genes
LBGs: Late biosynthetic genes
EBGs: Early biosynthetic genes
Cy: Cyanidin
