# Supplementary material for: Transcriptional regulation of proanthocyanidin biosynthesis pathway genes and transcription factors in Indigofera stachyodes Lindl. roots
Source: BMC Plant Biol. 2022 Sep 13;22:438. doi: 10.1186/s12870-022-03794-4 (PMC9469613; doi:10.1186/s12870-022-03794-4)
Supplement: Supplementary file 3 — Additional file 3. [file 12870_2022_3794_MOESM3_ESM.docx]

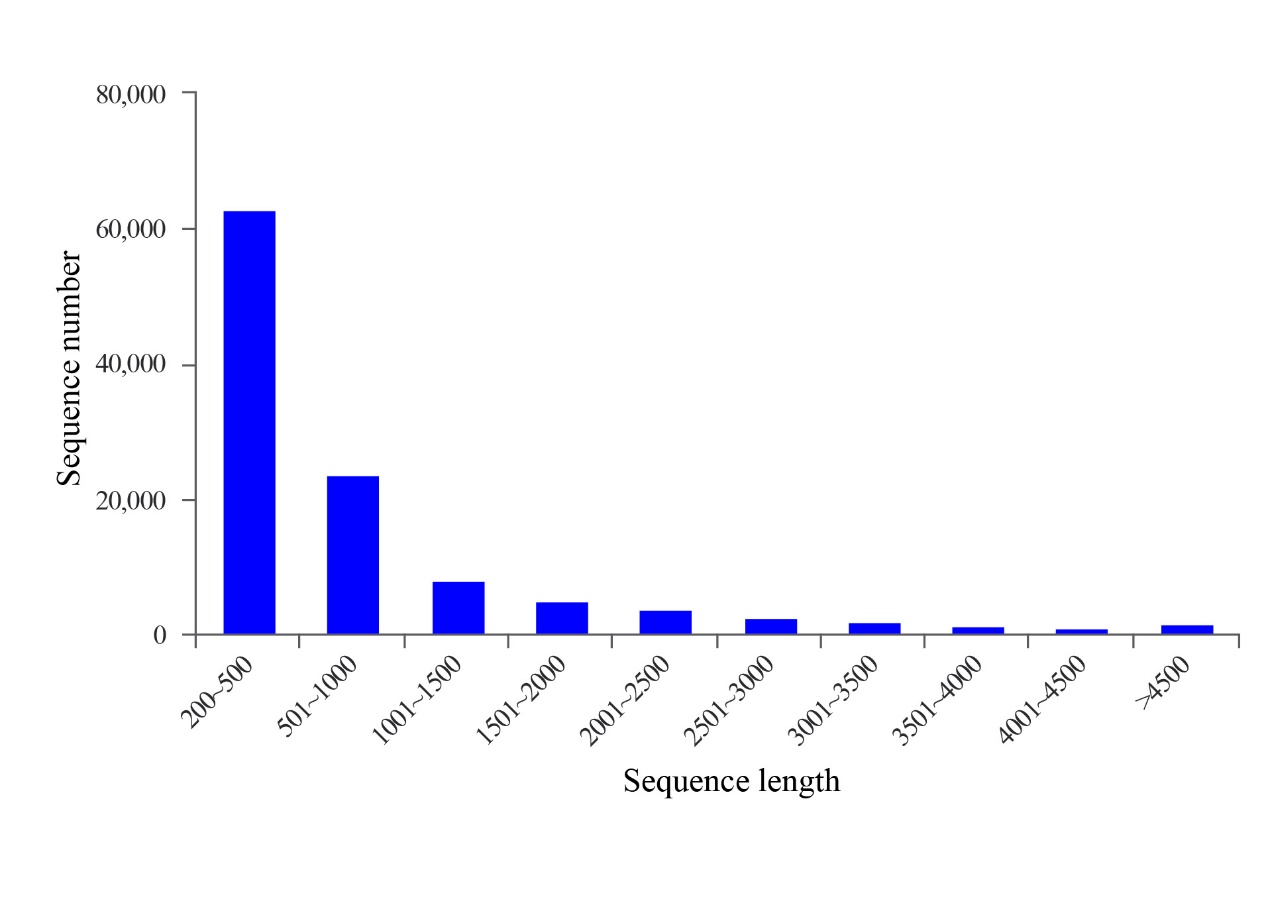


**Figure S1** Size distribution of the assembled unigenes.


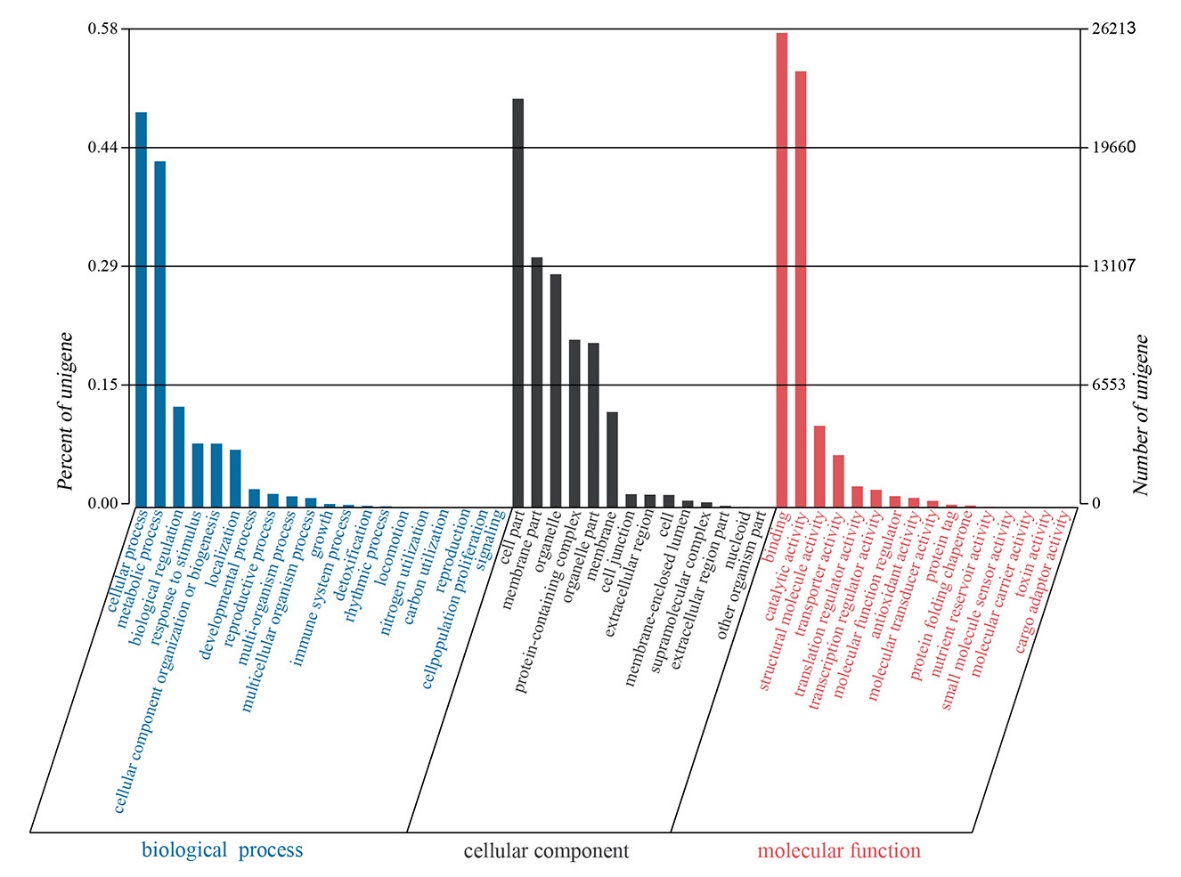


**Figure S2** Gene Ontology classification of assembled unigenes. The unigenes were categorized into three main categories: biological process, cellular component, and molecular function.


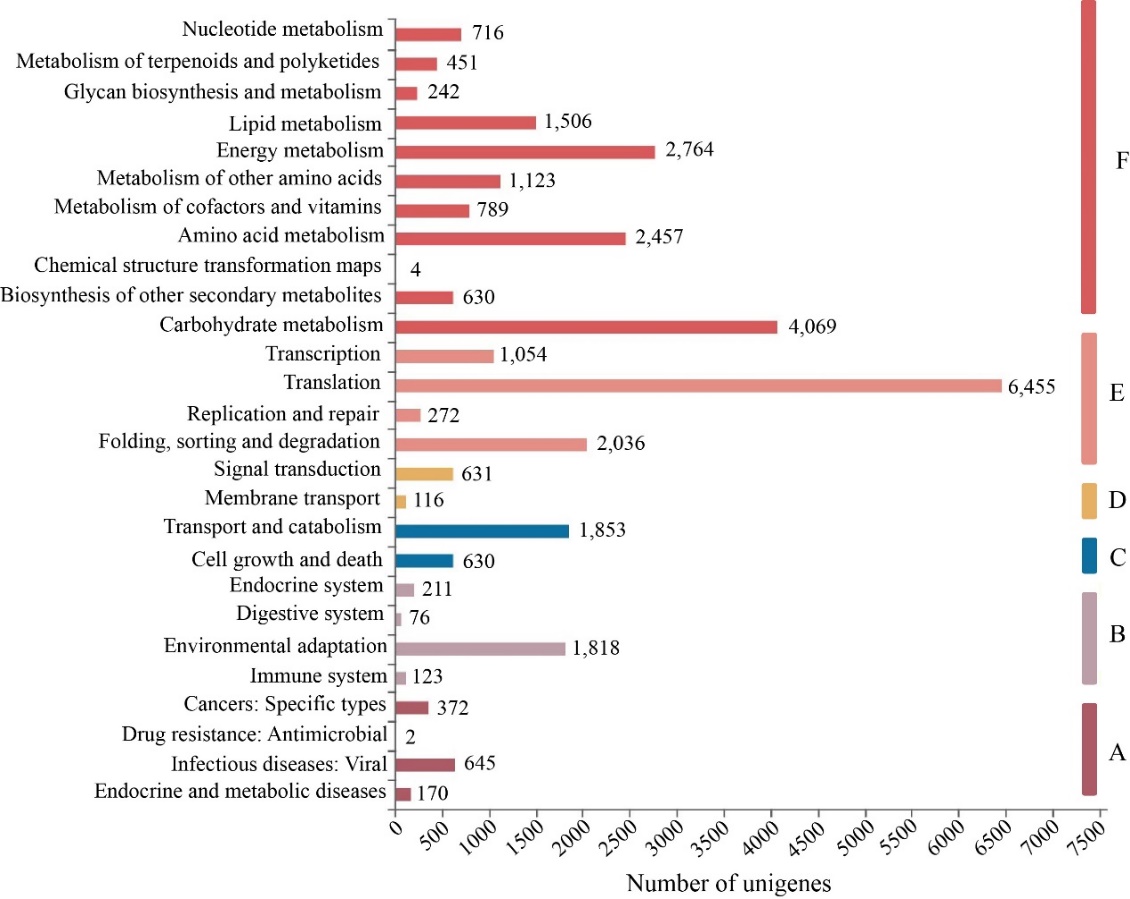


**Figure S3** Pathway assignment based on the Kyoto Encyclopedia of Genes and Genomes (KEGG). (A) Classification based on human diseases categories, (B) Classification based on organismal systems categories, (C) Classification based on cellular processes categories, (D) Classification based on environmental information processing categories, (E) Classification based on genetic information processing categories, and (F) Classification based on metabolism categories.


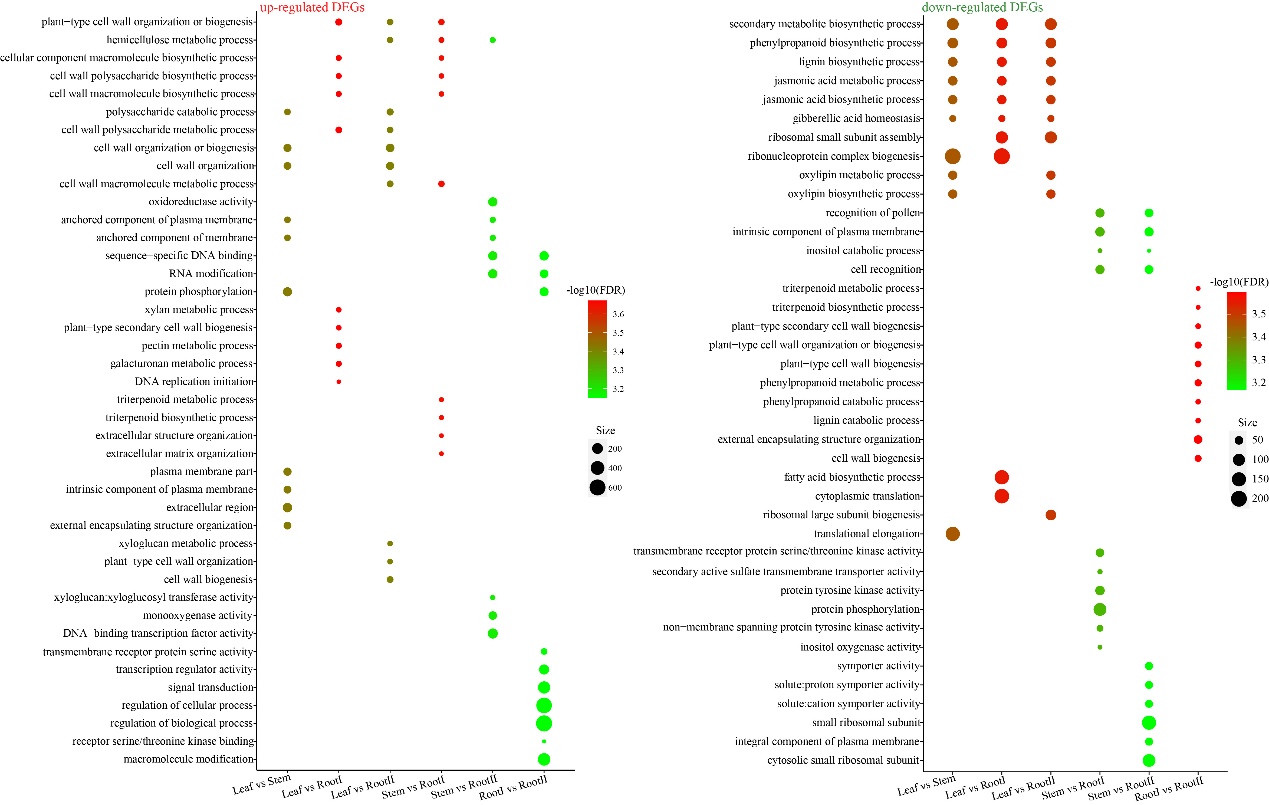


**Figure S4** The top 20 enriched GO terms of DEGs. The y axis shows the metabolic pathway terms, and the x axis shows the different comparison groups. The size of the plotted circle indicates the Sample number in this GO Term. The fill color is scaled to the -log10(FDR). (FDR < 0.05).

**
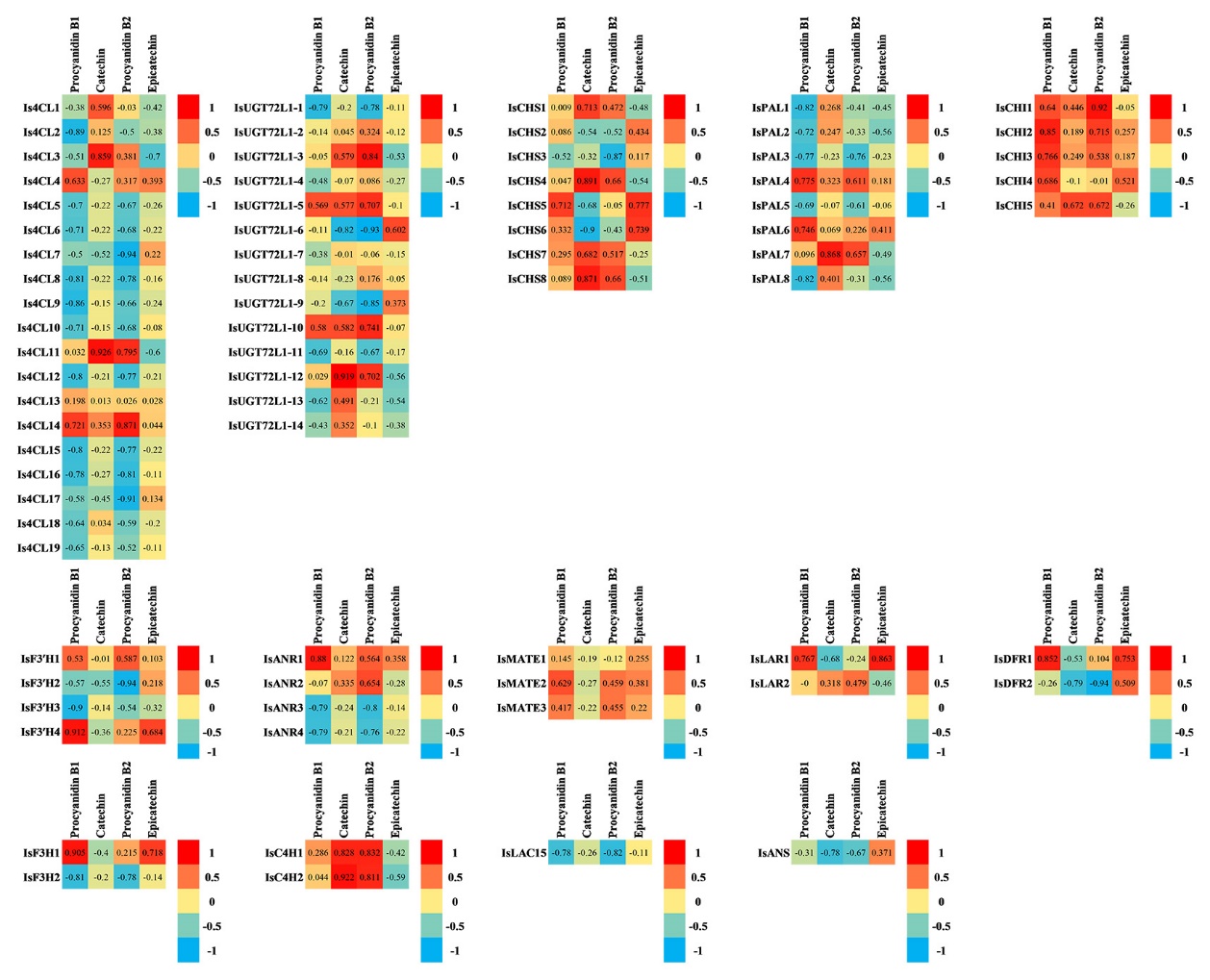
**

**Figure S5** PCC analysis of the correlation between structural genes and PAs. The color represents r value, which shows high positive correlation in red and high negative positive correlation in blue, while low correlation in yellow.


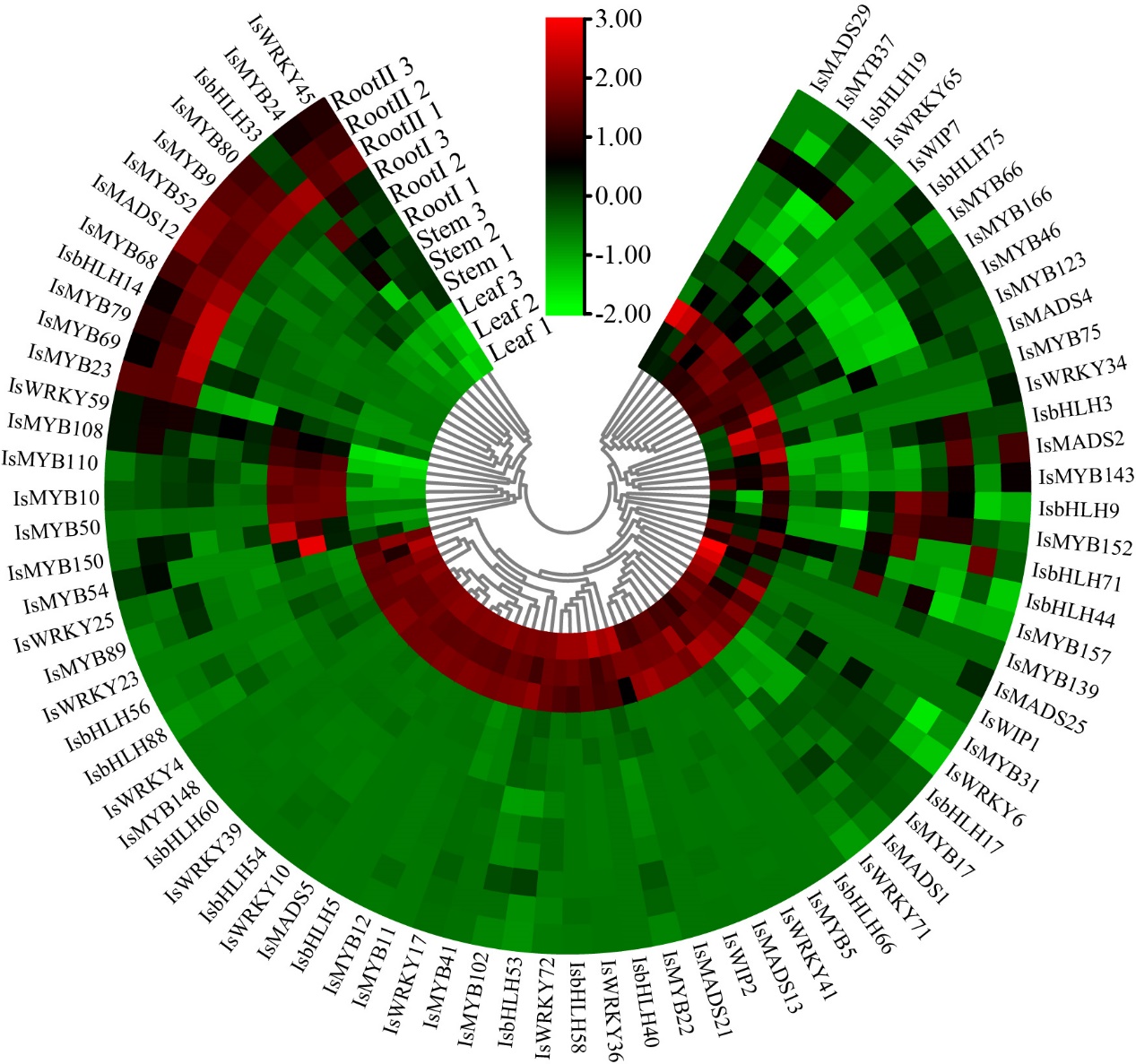


**Figure S6** Combinatorial TFs (MYB, bHLH, WRKY, MADS, WIP) expression across organ type. red is high expression, blue is low expression.


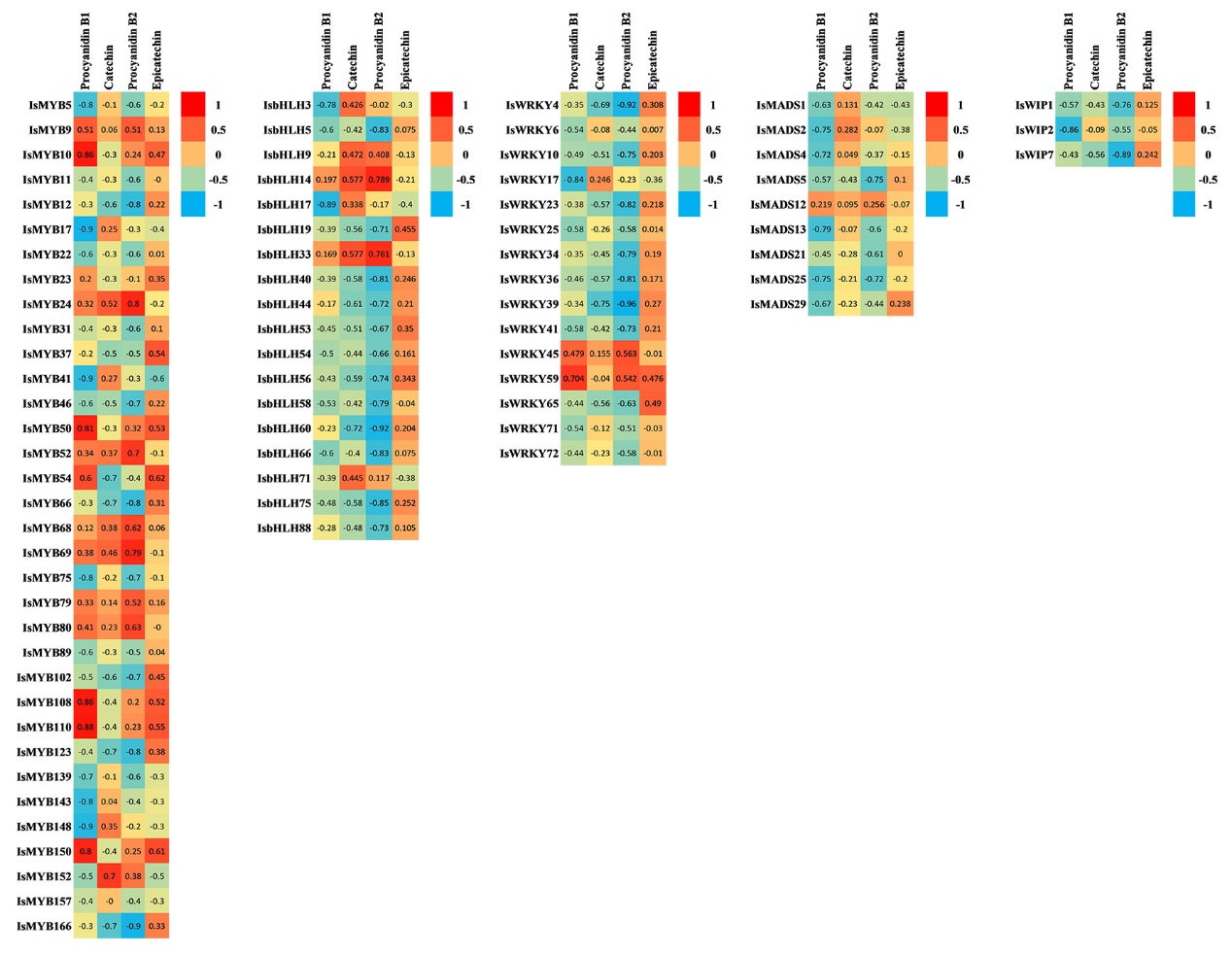


**Figure S7** PCC analysis of the correlation between transcriptional factors and PAs. The color represents r value, which shows high positive correlation in red and high negative positive correlation in blue, while low correlation in yellow.
